# Targeting extracellular matrix stiffness for cancer therapy

**DOI:** 10.3389/fimmu.2024.1467602

**Published:** 2024-12-02

**Authors:** Xiuqin Feng, Fujun Cao, Xiangji Wu, Wenyan Xie, Ping Wang, Hong Jiang

**Affiliations:** ^1^ Department of Biotherapy, State Key Laboratory of Biotherapy and Cancer Center, West China Hospital, Sichuan University, Chengdu, Sichuan, China; ^2^ Department of Pancreatic Surgery, West China Hospital, Sichuan University, Chengdu, Sichuan, China

**Keywords:** mechanical properties, matrix stiffness, tumor microenvironment, immunotherapy, solid tumors

## Abstract

The physical characteristics of the tumor microenvironment (TME) include solid stress, interstitial fluid pressure, tissue stiffness and microarchitecture. Among them, abnormal changes in tissue stiffness hinder drug delivery, inhibit infiltration of immune killer cells to the tumor site, and contribute to tumor resistance to immunotherapy. Therefore, targeting tissue stiffness to increase the infiltration of drugs and immune cells can offer a powerful support and opportunities to improve the immunotherapy efficacy in solid tumors. In this review, we discuss the mechanical properties of tumors, the impact of a stiff TME on tumor cells and immune cells, and the strategies to modulate tumor mechanics.

## 1 Introduction

Advanced solid tumor patients have poor responses to surgical and conventional treatments (1, 2). The emergence of cancer immunotherapy has significantly increased both the quality of life and survival rates of patients. However, its efficacy in solid tumors has been hampered by significant obstacles such as immunosuppression and targeted delivery challenges (3).

Solid tumors possess unique tumor microenvironment (TME). The primary drivers of this tumor microenvironment include a highly fibrotic stroma and extensive infiltration of immunosuppressive cell populations (4, 5). Owing to the abundance of collagen, the dense fibrous stroma leads to high stiffness of the tumor tissue, in terms of mechanical properties. From a macroscopic perspective, the highly stiff extracellular matrix (ECM), which is equivalent to a physical barrier, which can block the delivery of anticancer drugs and the infiltration of immune killer cells, thus affecting the efficacy of immunotherapy. At the microscopic level, mechanical stiffness can involve signaling pathways that mediate cell mechanics and affect cell phenotypes, behaviors and functions to promote tumor progression. On the basis of these characteristics, we introduce the physical characteristics, especially ECM stiffness of the TME in solid tumors, and discuss how the increased tissue stiffness affects tumor cells as well as the immune cells. In addition, we explore the strategies for altering the tumor stiffness to improve cancer therapy.

## 2 The mechanical properties of the ECM

### 2.1 ECM stiffness and its key regulators

Stiffness, also known as the modulus of elasticity, is the resistance to deformation of a material in response to a force applied at a very slow rate (6). Stiffness is an inherent physical property of tissue and has been used as a diagnostic marker for several solid tumors (7) and a prognostic indicator (8, 9), such as breast cancer, pancreatic cancer, prostate cancer, and colorectal cancer.

**Figure 1:**
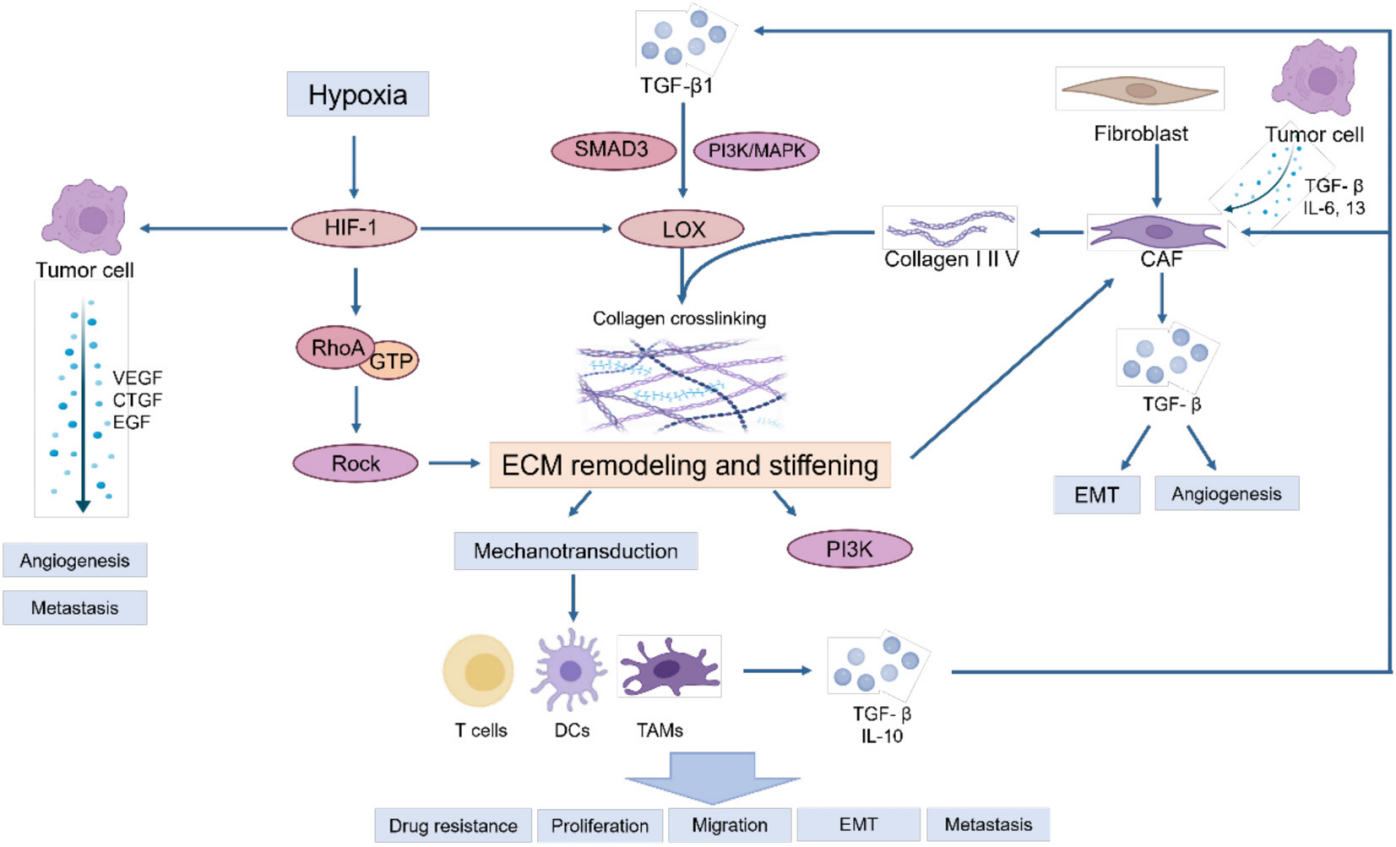
The primary causes of matrix stiffness and its effect on tumor microenvironment.

During the tumor initiation process, cancer cells release a range of growth factors, including TGF-β, IL-6, and IL-13, which play a crucial roles in the activation of fibroblasts into cancer-associated fibroblasts (CAFs) (10). CAFs are the main contributors to ECM deposition. CAFs are believed to originate from normal resident fibroblasts or quiescent stellate cells. They gradually transform into CAFs when stimulated by chemokines and cytokines (11–13). These activated fibroblasts have enhanced capabilities to synthesize and secrete ECM components. They promote the synthesis of collagen I, II, and V, and the assembly of collagenous fibers, thus remodeling the ECM and increasing tumor stiffness (14). On the other hand, tumor associated macrophages (TAMs) can induce the reprogramming of fibroblasts to CAFs by releasing TGF-β (5). Owing to excessive cell proliferation and tumor growth, the core region becomes hypoxic, thereby inducing the stable expression of HIF-1 (15, 16). Tumor cells, CAFs and TAMs activate LOX and transglutaminases in response to hypoxia and promote the assembly and cross-linking of collagenous fibers with the participation of cross-linkers such as fibronectin and tenascins, resulting in the deposition of large amounts of collagen and ECM proteins, leading to increased stiffness of the ECM (17). Moreover, stiffness activates TGF-β signaling (18, 19) and downstream Smad3, PI3K/MAPK and other signaling pathways to activate LOX to induce ECM remodeling (5, 20). Meanwhile, proteoglycans play a crucial role in ECM organization, cell adhesion, and signaling. In the ingredient of the tumor microenvironment, proteoglycans contribute to the physical barrier created by the ECM, influencing drug delivery and immune cell infiltration (21). Additionally, proteoglycans can bind to and modulate the activity of cytokines and growth factors, including inflammatory cytokines. This can create gradients of inflammatory cytokines within the tumor microenvironment, influencing the recruitment, activation, and polarization of immune cells (22, 23). These activities ultimately result in epithelial mesenchymal transformation of tumor cells, tumor cell migration and invasion, immune escape and therapeutic resistance (**Figure 1**).

Owing to the excessive proliferation of tumors, hypoxia in the core region induces the stable expression of HIF-1, activating and accelerating the synthesis of intracellular lysine oxidases (LOXs) and transglutaminases, especially LOX-1, LOXL-2 and transglutaminase-2, which further increases ECM stiffness (17).

Within this rigid and hydrated ECM network, various soluble factors are stored, such as growth factors, angiogenic factors, and chemokines, are stored, which collectively trigger a sustained inflammatory milieu. This inflammatory environment further promotes the generation of myofibroblasts and macrophages, leading to the deposition of significant amounts of growth factors and ECM proteins. Consequently, this process escalates the stiffness of the ECM, perpetuating a dynamic cycle of ECM remodeling and reinforcement (24, 25).

### 2.2 Cell response to increased mechanical stiffness

Tumor cells, immune cells and other cells share conserved pathways to sense and respond to mechanical cues. Many cell adhesion molecules, which are crucial for cell-matrix interactions and cell-cell communication, can function as mechanosensors, including integrins, selectins, and cadherins (26). Additionally, mechanosensitive ion channels that regulate the passage of ions such as Ca^2+^, Na^+^, and K^+^ also act as mechanosensors (27). For example, Piezo1 has been identified as a key mediator in the deletion of mechanical signals in both macrophages and T cells (28). Moreover, within lymphocytes, T cell receptors (TCRs) and B cell receptors (BCRs) play a critical mechanical roles in antigen recognition and the initiation of effector functions (29–31). These mechanosensors transmit signals that result in Ca^2+^ flux and the assembly of actin filaments (32, 33), which activate myosin to generate traction force. The active myosin assembles with filamentous actin and forms the skeleton of the actomyosin filament bundle.

Traction is transmitted along the chain of protein molecules to the ECM, which generates counterforces to balance the traction generated by myosin (34). The actin filament skeleton links the cell nuclear membrane to the linker of the nucleoskeleton and cytoskeleton (LINC complex) to transduce traction into the nucleus, activate YAP/TAZ to promote nuclear expression, and ultimately regulate gene and protein expression as well as cell phenotypes (35).

When the traction force reaches a certain threshold, some structural proteins are activated successively (36). First, talin exposes the active site and binds to the N-terminus of FAK, resulting in rapid phosphorylation of Tyr397. Activated FAK binds to a variety of downstream molecules and activates downstream RhoA and ROCK (37), which transmit signals to the nucleus and ultimately promote collagen synthesis of cancer associating fibrobrasts, leading to matrix remodeling and stiffening. These processes, which involve the conversion of cellular mechanical signals into biochemical signals, are known as mechanical transduction (38).

### 2.3 Other mechanical cues of the TME

There are other physical characteristics of the tumor, including solid stress (compression and tension), interstitial fluid pressure, and physical microstructure characteristics (6), in which abnormal changes contribute to tumor progression and resistance to treatment (39).

#### 2.3.1 Solid stress

Solid stress is the mechanical force (compression, stretching, and shearing) contained in the ECM and cells and is transmitted through solid and elastic elements. The solid stress increases with increasing tumor size. The increase in tissue volume is a result of cell infiltration, cell proliferation, and matrix deposition. This augmented volume exerts pressure, consequently generating solid stress in the tumor and surrounding tissues. Helmlinger et al. first proposed the effect of solid stress on cancer cell biology and reported that accumulated solid stress inhibited the growth of tumor spheres (40). These pressures are large enough to compress or even destroy the blood and lymphatic vessels (41–43). Vascular compression leads to hypoxia (43, 44) and interferes with the efficacy of radiotherapy, chemotherapy and immunotherapy (45–47). Solid stress may also have additional direct effects on tumor biology, such as promoting the aggressiveness of cancer cells (48) and stimulating tumorigenic pathways in the colon epithelium (49). Elevated solid stress can regulate fluid stress by compressing the blood and lymphatic vessels within the tumor. Vascular compression reduces tumor perfusion, whereas compression of lymphatic vessels impedes the tumor’s ability of tumors to expel excess fluid from the interstitial space, resulting in an even increase in interstitial fluid pressure (50).

#### 2.3.2 Interstitial fluid pressure

Normal interstitial fluid pressure homeostasis generally involves blood entering through arteries and veins, blood arriving through veins through arteries and lobes, and excess tissue fluid being expelled through lymphatic vessels. The presence of tumor tissue disrupts this homeostasis, showing high resistance to blood flow, low resistance to transcapillary fluid flow, and impaired lymphatic discharge (45, 51), resulting in increased interstitial fluid pressure (IFP). A high IFP hinders drug penetration to tumor sites, reduces the utilization rate, increases drug resistance, and affects the efficacy of radiotherapy, chemotherapy and immunotherapy (52).

#### 2.3.3 Tissue microstructure

In the human body, from every organ to every cell, there is a specific arrangement of microstructures, and constant normal evolution optimizes the stability, efficiency and function of tissues. Pathological changes disrupt these microstructures, leading to disturbances in homeostasis and facilitating the onset of various diseases. For example, the occurrence of atherosclerosis prevents the normal flow of blood, possibly leading to a lack of normal fluid shear force, eventually resulting in changes in the morphology and function of endothelial cells, and promoting vascular proliferation (53). Throughout the epithelial-mesenchymal transition process, epithelial cells undergo a loss of cell polarity. This results in a shift from the epithelial phenotype, which is anchored to the basement membrane, to a mesenchymal phenotype characterized by enhanced invasion and migration capabilities (54). In addition, excessive tumor growth eventually leads to abnormal collagen cross-linking, persistent stiffening of the stroma, and alterations in the TME, and ultimately facilitating immune evasion and resistance to therapy (55).

## 3 Matrix stiffness regulates the tumor microenvironment

### 3.1 Effects of matrix stiffness on tumor cells

#### 3.1.1 Increased tumor cell proliferation and survival

A stiff ECM activates signaling pathways, such as the FAK, MAPK, and PI3K/Akt pathways in tumor cells, and enhances their proliferation and survival capabilities (56, 57).

#### 3.1.2 Promotion of tumor cell migration and invasion

Increased matrix stiffness can promote the migration and invasion of tumor cells, making it easier for them to penetrate the ECM and enter blood or lymph vessels, leading to tumor metastasis (58, 59). Stiffness-mediated downregulation of the antiangiogenic isoform of VEGF, which results in the alternative splicing of more proangiogenic isoforms (60), could play an important role in regulating angiogenesis. The presence of laminin β1 chains in the ECM increases cell–cell contact during tube formation (61). In contrast, collagen I ensures the disruption of cell–cell connections (62). Notably, since collagen I fis the main component of many surrounding tissues, it promotes the migration behavior of the cells.

### 3.2 Effects of matrix stiffness on the behavior of immune cells

Interestingly, within the TME, particularly in solid tumors, immune cells experience comparable physical and mechanical conditions characterized by specific pressure and stiffness. Research has focused primarily on elucidating the impact of various biochemical signals on immune cells (63, 64), and the specific implications and underlying mechanisms of mechanical stiffness on immune cell behavior remain unclear. Here, we summarize the influence of physiologically related mechanical cues on the polarization, function, and activity of various immune cells.

#### 3.2.1 Monocytes

Monocytes and their derived macrophages, which are involved in tissue repair and remodeling, are responsive to various mechanical microenvironments. The expression of proinflammatory genes and cytokines, such as NOS2, IL-12β, IL-6, and IL-8, is upregulated in human monocytes encapsulated in agarose and exposed to a combination of shear and compression conditions. Monocyte activation tends to be associated with more M1-like phenotypes, highlighting the response of human monocytes to mechanical stimuli (65).

Hypertension and endothelial mechanical stretching have been reported to regulate the phenotype and function of monocytes. Coculture of human monocytes with fused human aortic endothelial cells under cyclic stretching of 5% or 10%, similar to hypertension, affected the distribution of human circulating monocytes, with the percentage of classical monocytes decreasing and the proportion of intermediate and nonclassical monocytes increasing. Additionally, the expression of IL-6, IL-1β, IL-23, CCL4 and TNF-α were increased in monocytes, leading to the promotion of monocyte differentiation and activation (66).

#### 3.2.2 Macrophages

Macrophages are among of the most predominant immune cells within the TME and promote tumor growth and immune suppression (67, 68). They facilitate tumor growth by promoting neoangiogenesis, remodeling the matrix, and inhibiting tumor immunity and other mechanisms (67). While they promote ECM remodeling, macrophages are affected by changes in substrate stiffness. In addition, macrophages exhibit mechanical sensitivity to the hardening mechanical microenvironment and can respond to variations in matrix stiffness by altering the area, phenotype, migration rate and mode, function and actin tissue regulation. On rigid (150 kPa) substrates, the stiffness and phagocytosis capacity of macrophages increase, and further studies have indicated that the function of macrophages is regulated by actin polymerization (68).

Studies have shown that substrate stiffness strongly influences the phagocytic function and polarization phenotype of macrophages, and the specific influence is mainly determined by the origin of the macrophages, the biomaterial model used and the different chemical stimuli used (63, 68–71). Rukmani Sridharan et al. showed that the phagocytosis and migration of macrophages were impaired by stiff gel, and the migration mode was mainly mesenchymal, which was different from that of a softer matrix, and involved mainly ROCK-dependent deformation (63). Cougoule et al. suggested that there were differences in the migration patterns of macrophages in different substrate forms, such as amoeba migration in a porous matrix (e. g., fibrillary collagen type I) and mesenchymal migration in a dense matrix (e.g., matrix gel) accompanied by matrix proteolysis (72). Moreover, the migration rate of human monocyte-derived macrophages on flat fibronectin-coated PA gel was positively correlated with stiffness (73).

#### 3.2.3 T cells

T cells are mechanically sensitive throughout their life cycle and are always exposed to different mechanical microenvironments, ranging from soft tissues such as the thymus or bone marrow to rigid tissues such as inflammatory and tumor tissues, which affects the function of T cells.

TCRs act as essential mechanical sensors in T cells, and mechanical forces initiate TCR signaling by acting directly on the TCR complex rather than on other surface receptors (74–76). ECM stiffness can greatly increase the activation of T cells (29) by extending the lifetime of the ligand-T cell receptor (TCR) bond, and triggering the influx of calcium ions (77, 78).

The migration, cytokine secretion, metabolism and cell cycle progression of human CD4^+^ T cells vary with substrate stiffness (76, 79). Migration was monitored on PA-hydrogels of various stiffness coated with biotinylated ICAM-1. CD3 and CD28 antibodies were used to activate T cells. The mean instantaneous velocity and migration distance of T cells on 100 kPa PA gels were significantly greater than those on 0. 5 kPa and 6. 4 kPa gels. With increasing rigidity, TCR/CD3, the main rigid sensing receptor, induced stronger signal transmission and gene expression. The expression of cytokines, T-cell surface markers, T-cell-specific transcription factors (TBX21 and Foxp3), and the proliferative transcription factor MYC increased with increasing stiffness in the presence of the CD3. However, it is interesting to note that some T-cell functions such as cytokine signaling, and T-cell activation, can be triggered at a lower stiffness value range (0.5– 6.4 kPa), whereas others (respiratory electron transport and glycolysis) require greater stiffness (6.4 –100 kPa). Moreover, the TCR activation induced glycolytic metabolism, the cell cycle, and the proliferation of T cells increase in response to stiff substrates (79).

The induction of human Treg cells is mechanosensitive and dependent on oxidative phosphorylation (OXPHOS). Specifically, Treg induction and metabolism are enhanced on stiffer substrates (80). In aged skin, a more aligned ECM resulting from the loss of the hyaluronic and proteoglycan link protein HAPLN1 impedes CD8^+^ T cell migration while promoting Treg infiltration in melanoma (81). Additionally, activation of integrin α4β1 has been shown to enhance the immunosuppressive capacity of Treg cells, whereas the loss of talin—an integrin-binding protein—can lead to severe systemic autoimmunity (82). Notably, collagen, a primary component of the ECM, can increase the expression of Treg biomarkers such as CD4, FOXP3, and CD25, thereby supporting the immunosuppressive TME (83).

Furthermore, the inducible co-stimulatory molecule (ICOS), a member of the CD28/CTLA4 family, is expressed on activated T cells (84, 85). ICOS-mediated costimulation facilitates the production of cytokines such as IL-4 and IL-10, suggesting the role of ICOS in supporting secondary, memory, and effector T cell responses (86, 87).

#### 3.2.4 B cells

B cells sense antigens through B-cell receptors (BCRs) and react differently, contributed by the varying rigidity of antigens presented on substrates (88, 89). For example, virus particles exhibit greater stiffness (45–1,000 MPa) (90), most mammalian cells have medium stiffness (0.01–1,000 kPa) (91), and the secreted soluble pathogen antigens have a low stiffness of less than 100 Pa (92). Substrate stiffness guides B cell activation, proliferation, class switching and the antibody response *in vivo* (88, 93).

#### 3.2.5 Dendritic cells

Dendritic cells (DCs), specialized immune cells, scan for foreign bodies or abnormal cells in the surrounding tissue. Once they recognize this danger signal, they travel to the lymph nodes to activate T cells and then trigger an immune response. To do this, dendritic cells travel long distances in the body and encounter a variety of microenvironments with different mechanical properties, such as tissue stiffness (94).

Studies have shown that mechanical stiffness is a key physical cue affecting DC differentiation/maturation, phenotype, metabolism, quality and function (94, 95). The expression of C-type lectins on immature DCs (IDCs) is regulated by substrate rigidity, leading to the internalization of different antigens. In addition, substrate rigidity impacts β-2 integrin expression and foot formation in IDCs, thus affecting cell functions (94).

DCs respond to increased stiffness through both functional and metabolic reprogramming. Higher stiffness upregulated glucose metabolism in DCs to support their inflammatory phenotype. Stiffness bolstersBMDC differentiation *in vitro*. Tension effects on DCs are potentially transient and reversible, to allow for the regulation of DC activation. In the course of immunotherapy, high-tension DC cells enhance the main adaptive immune capacity for tumor clearance. This effect does not require pattern-recognition receptor (PRR) ligation. In addition, rigid substrates can result in crosstalk of the adaptive immune system, contributing to diseases such as diabetes and pancreatitis. A major hippo signaling factor, TAZ, and a mediator of ion homeostasis, including PIEZO1, a potential tension sensor in DCs, regulate the metabolic function of DCs. Tension also influences the phenotype of human monocyte-derived DCs (95).

## 4 ECM stiffness affects the immune therapy response

### 4.1 Stiffness of the ECM affects drug infiltration

Many immunotherapies, such as immune checkpoint inhibitors, cancer vaccines, and tumor microenvironment modulators, exert their effects through drugs. The physical and biochemical properties of the tumor ECM can influence the ability of drugs to enter and diffuse within the tumor microenvironment. In particular, the stiffness of the ECM plays a significant role in the infiltration and delivery of drugs. As the tumor progresses, the ECM becomes dense and rigid. This rigidity and compactness act as a physical barrier, impeding the effective infiltration of drugs (96). The cross-linking and accumulation of collagen and other fibers within the ECM might restrict the penetration of drug molecules (97). Moreover, the increased stiffness of the ECM alters the morphology and function of tumor microvessels, thereby affecting the transport of drugs from blood vessels to tumor tissue (98). Additionally, the increased stiffness and density of the ECM can increase interstitial fluid pressure, which might further hinder the invasion and dispersion of drugs. Owing to this elevated hydrostatic pressure, the driving force for drugs to move from blood vessels into the tumor tissue may decrease (99).

### 4.2 The stiffness of the ECM mediates the formation of an immunosuppressive microenvironment

Increased stiffness of the ECM can promote the formation of an immunosuppressive TME in various ways. The increased stiffness of the ECM can act as a physical barrier, limiting the migration and infiltration of immune cells, such as T cells, NK cells, and macrophages, thereby reducing their presence within the tumor tissue (100–102). The stiffness of the ECM can influence the activation and differentiation of immune cells through mechanical signaling pathways. For instance, T cells experience reduced stimulation in a high-stiffness ECM, leading to their functional suppression (63). Increased ECM stiffness can also favor the differentiation of certain immune cells, such as macrophages, toward an immunosuppressive phenotype. Variations in ECM stiffness may promote the accumulation of regulatory T cells (Tregs) and myeloid-derived suppressor cells (MDSCs) in the tumor microenvironment (103). The immunosuppressive environment mediated by ECM stiffness substantially hampers the application of adoptive cell therapies, such as CAR-T cells, in solid tumors (104).

### 4.3 ECM stiffness affects the immune cells

ECM stiffness represents a significant factor driving macrophage polarization toward the M2 phenotype. A notably higher proportion of M2-like macrophages was identified in the stiffer ECM of mouse mammary tumor by single-cell RNA sequencing (105). It has been reported that CAFs are highly correlated with tumor-associated macrophages. In patients with poorer clinical prognosis, there is a concomitant high expression of both CAF and TAM markers, such as α-SMA, FAP, and CD163 (106, 107). Furthermore, CAFs are able to facilitate monocyte migration into tumors and polarize into the M2 phenotype. For instance, CAF-derived M-CSF1, IL-6, and CCL2 in monocyte recruitment increased the M2/M1 TAM ratio in pancreatic cancer (108).

ECM stiffness affects T cell migration and infiltration. One study found that T cells stranded in condensed fibrotic collagen areas surrounding human hepatocellular carcinoma highly express both PD-1 and TIM-3, markers for late exhausted CD8^+^ T cells. It suggested that high environmental stiffness can promote CD8^+^ T cell exhaustion (109). In 20 resected triple-negative breast cancer samples, immunostaining for CD8 and picrosirius red staining for fibrous collagen were performed. The results showed that samples with high collagen density usually had fewer infiltrating CD8^+^ T cells (110). In mice models, in soft tumors those induced by LOX inhibition, T cells are able to migrate. On the contrary, in stiff non-treated control tumors, T cell migration is hindered (111).

The implications of ECM stiffness also extend to other immune cell types. The protein STEAP3, whose activity is influenced by matrix stiffness, facilitates neutrophil infiltration (112). Additionally, SOX9, by increasing collagen deposition and thereby intensifying ECM stiffness in Kras^+^G12D-driven murine LUAD, leads to reduced infiltration of DCs within tumors, thereby suppressing CD8^+^ T cell and NK cell infiltration and activity (102).

### 4.4 ECM architecture changes affect immune therapy

In lung cancer, high collagen correlates with reduced anti-PD-1/PD-L1 efficacy. Anti-PD-L1 resistance in lung cancer mouse models is associated with enhanced collagen deposition and fewer exhausted tumor-infiltrating CD8^+^ T cells. Therapeutic targeting of the LAIR-1 pathway in tumor models promoted the activation and function of T cells, NK cells, macrophages, and DCs (113). Blockade of LAIR-1 enhances anti-PD-L1 efficacy (114–116). Blockade of LAIR-1 also works in humanized murine xenograft models (115, 117). LOX-inhibitor reduces tumor stiffness, increases tumor-infiltrating T cells, and improves anti-PD-1 response (111). Inhibition of FAK in pancreatic cancer murine models reduces collagen deposition, decreases anti-inflammatory immune cells, increases CD8^+^ T cells, and improves the efficacy of TIL-based and checkpoint inhibitor therapy (118). A bacterial-based agent delivering collagenase to murine pancreatic tumors reduces collagen levels and enhances checkpoint inhibitor treatment (119).

Overexpression of matrix metalloproteinases (MMPs) is associated with a poor prognosis in cancer. Anti-MMP-9 treatment can increase certain T cell-related factors, including IL-12p70 and IL18 (120). MMP2/9 is correlated with tumor-infiltrating lymphocytes (TILs), T cell exhaustion, and inhibitory immune checkpoints. An MMP2/9 inhibitor called SB-3CT enhances T cell-mediated cytotoxicity. Moreover, SB-3CT improves the efficacy of anti-PD-1 and anti-CTLA4 treatment in mouse models of melanoma and lung cancer as well as in metastatic melanoma in the lung (121). Knockdown of MMP-1 expression in TNBC cells inhibits breast cancer growth and brain metastasis in a xenograft model (122). MMP-1 is also involved in tamoxifen resistance in breast cancer. Downregulation of MMP1 in tamoxifen-resistant breast cancer cells induces tamoxifen sensitivity *in vitro* and retards tumor growth *in vivo* (123).

## 5 Substrate mechanics: potential therapeutic targets and drugs

As we mentioned above, increased ECM stiffness has been associated with increased cancer cell proliferation, enhanced cell survival, and the induction of an immunosuppressive microenvironment. Addressing this stiffness through targeted therapies may enhance the penetration of drugs into tumor cells, potentially improving treatment outcomes. On the other hand, ECM degradation has been correlated with cancer cell migration, invasion, and the angiogenesis induction (124). Therefore, a meticulously planned strategy to target both ECM stiffness and processes such as cell migration and angiogenesis is paramount for optimizing therapeutic effectiveness while minimizing unintended side effects. Here we focus on strategies that target ECM stiffness (**Table 1**).

**Table 1:** Targeting ECM components.

Target	Drug name	Category	Stage	ClinicalTrials.gov ID	Diseases
Myofibroblasts	MRG-201	MicroRNA	Phase I	NCT02603224	Fibrous scar
TGF-β	pirfenidone	Small molecule inhibitor	Phase II, III	NCT03068234	Skin Fibrosis
			Phase II, III	NCT01933334	Systemic Sclerosis
			Phase III	NCT01366209, NCT00287729, NCT00287716	Idiopathic Pulmonary Fibrosis
	Losartan	Angiotensin-receptor blocker	Phase II	NCT01821729	Pancreatic Cancer
	Lucan ix™	Antisense gene-modified allogeneic tumor cell vaccine	Phase III	NCT00676507	Lung Neoplasm, Non-small Cell Lung Cancer Stage IIIA, IIIB, IV
	Nintedanib	Small-molecule tyrosine kinase inhibitor	Phase II	NCT01170065	Pulmonary Fibrosis
			Phase II	NCT02389764	HER2-Negative Metastatic Inflammatory Breast Cancer
CTGF	Pamrevlumab (FG-3019)	Monoclonal antibody	Phase I, II, III	NCT01262001, NCT00074698, NCT01890265, NCT04419558, NCT03955146	pulmonary fibrosis
			Phase I	NCT01181245	Locally Advanced or Metastatic Pancreatic Cancer
			Phase II	NCT02047513	Resectable Pancreatic Cancer
LOX	Simtuzumab (GS-6624)	Monoclonal antibody	Phase I, II	NCT01769196, NCT01362231	Idiopathic Pulmonary Fibrosis
			Phase II	NCT01479465	Metastatic KRAS Mutant Colorectal Adenocarcinoma
			Phase II	NCT01472198	Pancreatic Cancer
			Phase II	NCT01369498	Myelofibrosis
	Tetrathiomolybdate	Copper chelator	Phase II	NCT00195091	Breast carcinoma
			Phase I, II	NCT00189176	Idiopathic Pulmonary Fibrosis
			Phase III	NCT00805805	Primary Biliary Cirrhosis
			Phase I	NCT01837329	NSCLC
	GB2064	Small molecule inhibitor	Phase II	NCT04679870	Myelofibrosis

### 5.1 Targeting ECM components to reduce mechanical stiffness

#### 5.1.1 Myofibroblasts

Preclinical experiments have shown that targeting myofibroblast apoptosis is an effective antifibrotic treatment. The monoclonal antibody C1-3 specifically targets transmembrane proteins expressed by hepatic myofibroblasts, and when combined with gliotoxin, it can induce the apoptosis of myofibroblasts and significantly reduce the severity of fibrosis (125). In addition, the abnormal transformation of fibroblasts into myofibroblasts can be inhibited. In this process, signals such as reactive oxygen, microRNAs, chemokines, and cytokines, which are important mediators of the phenotypic transformation of myofibroblasts, can be used as potential therapeutic targets (126–129) to inhibit the formation of myofibroblasts. MRG-201, a drug similar to miR-29, was recently tested in a phase 1 clinical trial for antifibrosis therapy (129) (NCT02603224). However, normal wound healing and other physiological functions require the critical involvement of myofibroblasts, and it has also been shown that depletion of myofibroblasts in the stroma leads to increased tumor aggressiveness and decreased survival (130), which is counterproductive.

#### 5.1.2 TGF-β

TGF-β is widely involved in the occurrence and development of fibrosis in different organs. TGF-β is also a well-studied profibrotic cytokine. During the initiation of fibrosis, the overproduction of TGF-β or the enhancement of its profibrotic effect leads to an abnormal wound healing response. Moreover, TGF-β within the TME has been implicated in promoting immunosuppression, promoting angiogenesis, and epithelial-mesenchymal transition (EMT) (131–133). By targeting TGF-β, not only can the ECM be remodeled, but the TME can also be optimized to facilitate more effective cancer therapy outcomes. At present, many drugs targeting TGF-β, such as drugs prepared from peptides, antisense oligonucleotides, small molecule inhibitors, monoclonal antibodies and vaccines, have been developed and have entered phase I, II, and III clinical trials (134).

The ability of pirfenidone to inhibit TGF-β has been confirmed in clinical trials (135, 136), and it was the first drug approved for idiopathic pulmonary fibrosis (IPF) treatment in Europe and was in phase III trials in the United States (134, 137, 138) (NCT01366209, NCT00287729, NCT00287716, and NCT01504334). Belagenumatucel-L is an antisense prepared as an enhanced tumor vaccine that is actually a genetically engineered non-small cell lung cancer tumor cell line with better activity than a conventional tumor vaccine vaccination (139, 140). Significantly increased survival was found in patients treated with each dose of this vaccine, who entered a phase II/III clinical trial (141) (NCT00676507). In addition, the angiotensin receptor 1 blocker losartan reduces collagen I and HA by inhibiting TGF-β (44). In a preclinical model of PDAC, the strategies of losartan in reducing solid pressure, decompressing blood vessels, enhancing chemotherapy, and improving overall survival are currently being tested in a randomized clinical trials (142) (NCT01821729).

#### 5.1.3 CTGF

CTGF mediates the expression and signaling of TGF-β, which circulates through the TGF-β pathway and subsequently induces additional TGF-β production (143). CTGF, which is essential for TGF-β-mediated fibrosis, binds directly to TGF-β to enhance receptor association (144). Therefore, CTGF promotes ECM remodeling and fibrosis pathology by indirectly regulating ECM synthesis and MMP expression in myofibroblasts.

FG-3019 is a full-human monoclonal antibody against CTGF. Preclinical studies have shown that FG-3019 can penetrate tissues and reduce the effective tissue level of CTGF, thereby reducing profibrotic factors, rebalancing ECM secretion and processing, and restoring tissue homeostasis (144, 145). FG-3019 has been evaluated for the treatment of pulmonary fibrosis and has shown good safety and tolerability, as well as good results in imaging changes in pulmonary function and the degree of pulmonary fibrosis (144, 146) (NCT01262001, NCT00074698, NCT01890265, NCT04419558, and NCT03955146). In preclinical trials, FG-3019 combined with gemcitabine was found to promote tumor stability and prolong survival, with better efficacy than any single treatment (147). Therefore, FG-3019 was added to gemcitabine and erlotinib in a follow-up study in which naive patients with locally advanced or metastatic pancreatic cancer were recruited, and the results revealed good safety with significantly better overall survival (148) (NCT01181245). In addition, the research results support that FG-3019 has a good effect on the treatment of skin fibrosis, but it has not entered into clinical trials (145).

#### 5.1.4 LOX

Lysyl oxidase (LOX), a typical member of five secretory copper-dependent enzyme families, promotes covalent cross-linking through the oxidative deamination of peptidyl lysine residues in collagen and elastin, thereby reshaping the stiff extracellular matrix (149, 150). LOX is now being recognized as a promising therapeutic target because of its dual involvement in the tumor stroma and premetastatic niche formation (151). β-Aminopropenitrile (β-APN) is a nonspecific, irreversible inhibitor of the lipoxygenase family that covalently binds to the active site of the lipoxygenase family of enzymes and is the first widely used LOX family inhibitor; however, its use was discontinued because of its high toxicity in clinical trials (152). GS-6624, a monoclonal antibody against LOX 2 (LOXL2), was well tolerated in the first half of the phase II safety study, but patients with cancer and fibrosis disease did not benefit from the phase II clinical trial (153–155) (NCT01362231, NCT01769196, NCT01479465, and NCT01472198).

In the race to create effective LOX inhibitors, a considerable challenge is that the complete crystal structure of mammalian LOX remains unknown (156). Therefore, other approaches have been proposed, including inhibition of LOX transcription factors or prevention of BMP-1 posttranslational cleavage of precursor peptides (156). The depletion of copper LOX catalytic sites in the nonspecific copper chelator tetraithiomolybdate reduced the serum LOXL2 concentration in patients with moderate- to high-risk primary breast cancer in a phase II clinical trial (157) (NCT00195091). Tetrathiomolybdate has also been used to treat idiopathic pulmonary fibrosis (NCT00189176), primary biliary cirrhosis (NCT00805805) and non-small cell lung cancer (NCT01837329). Overall, compared with β-APN, tetrathiomolybdate therapy is favorable because of its simple oral administration route, excellent tolerability, and stronger LOX inhibition (156, 157). PXS-5120A (158) and PXS-5153A (159), which are halinated allylamine drugs, have been shown to reduce collagen cross-linking, reduce the degree of liver and pulmonary fibrosis, and improve liver and lung function (158, 159). A phase I clinical trial of an oral LOXL2 inhibitor, PXS-5382A, was completed in healthy adults (NCT04183517). PAT-1251, a highly specific small molecule inhibitor of LOXL2 based on benzylamine and 2-substituted pyridine-4-methylamine, has not been tested in a phase II clinical trial to date, although it was found to be well tolerated and successfully passed a phase I clinical trial (NCT02852551). A preclinical study of the aminomethiophene-based LOX inhibitor CCT365623 demonstrated that inhibition of LOX led to delayed tumor development and reduced lung metastasis in a mouse model of breast cancer (160–162). Furthermore, CCT365623 has been shown to have good stability and specificity for LOX (160–162) but has not yet been tested in a clinical setting.

#### 5.1.5 MMPs

MMPs, which are secreted by tumor cells, stromal cells and other cells, play crucial roles in selectively cleaving ECM components. Their capacity to cleave and activate growth factors, chemokines, cytokines, and receptors is closely linked to the metastasis cascade and tumor angiogenesis processes, ultimately driving cancer progression (163, 164). MMPs can activate TGF-β, CTGF, KGF, macrophage inflammatory protein (MIP), bone morphogenetic protein (BMP), and other factors that are crucial for tumor progression and immune regulation. Additionally, MMPs can cleave proteoglycans, such as syndecans and glypicans, which are important for cell adhesion, signaling, and ECM organization. The release of these factors can further modulate the immune response and influence the infiltration and function of immune cells within the tumor microenvironment (165–167).

Efforts to target MMPs to combat cancer metastasis have been extensively pursued in clinical trials but have ultimately been proved unsuccessful in patients. Hence, strategies that involve modifying MMP activity to reduce ECM stiffness should be approached with caution (168, 169).

### 5.2 Blocking abnormal mechanical transmission signals

#### 5.2.1 Integrin

Integrins, as the connecting proteins, play direct connections from the matrix to the intracellular space (**Table 2**). In general, integrins serve as bridges for cell-ECM interactions, and their activity is influenced by ECM stiffness. As initiators of the mechanical sensing signaling pathway, activated integrins are able to activate myosin to generate force and then transduce mechanical signals to the nucleus to activate YAP/TAZ. Moreover, it can activate the mechanical sensor FAK and initiate downstream signaling cascades such as Src and Rho/ROCK, acting as a feedback loop to promote stromal stiffening and further worsen tumors (170). Preclinical studies have also shown that integrins can promote the progression of malignant tumors, including invasion, metastasis and drug resistance. Therefore, the specific targeting of integrins to directly block mechanical sensing in and out of cells is an attractive target.

**Table 2:** Blocking abnormal mechanical transmission signals.

Target	Drug name	Category	Stage	ClinicalTrials.gov ID	Diseases
Integrin	Etaracizumab (MEDI-522)	monoclonal antibody	Phase II	NCT00066196	Malignant Metastatic Melanoma
	CNTO95	monoclonal antibody	Phase I	NCT00888043	Solid Tumors
	Cilengitide	Small molecule inhibitor	Phase II	NCT00246012	Melanoma
			Phase I, II, III	NCT00103337, NCT00121238	Recurrent Prostate Cancer
	GSK3008348	Small molecule inhibitor	Phase I, II	NCT01118676, NCT00842712	NSCLC
			Phase II, III	NCT00813943, NCT00093964, NCT00689221	Glioblastoma
			Phase I	NCT03069989, NCT02612051	Idiopathic Pulmonary Fibrosis
FAK	PF-00562271	Small molecule inhibitor	Phase I	NCT00666926	Head and Neck Neoplasm Prostatic Neoplasm Pancreatic Neoplasm
	VS-6063	Small molecule inhibitor	Phase I	NCT01778803	Ovarian Cancer
	VS-4718	Small molecule inhibitor	Phase I	NCT01849744	Metastatic Non-Hematologic Malignancies
	GSK2256098	Small molecule inhibitor	Phase I	NCT01138033	Solid Tumors
			Phase II	NCT02428270	Pancreatic Cancer

**Figure 2:**
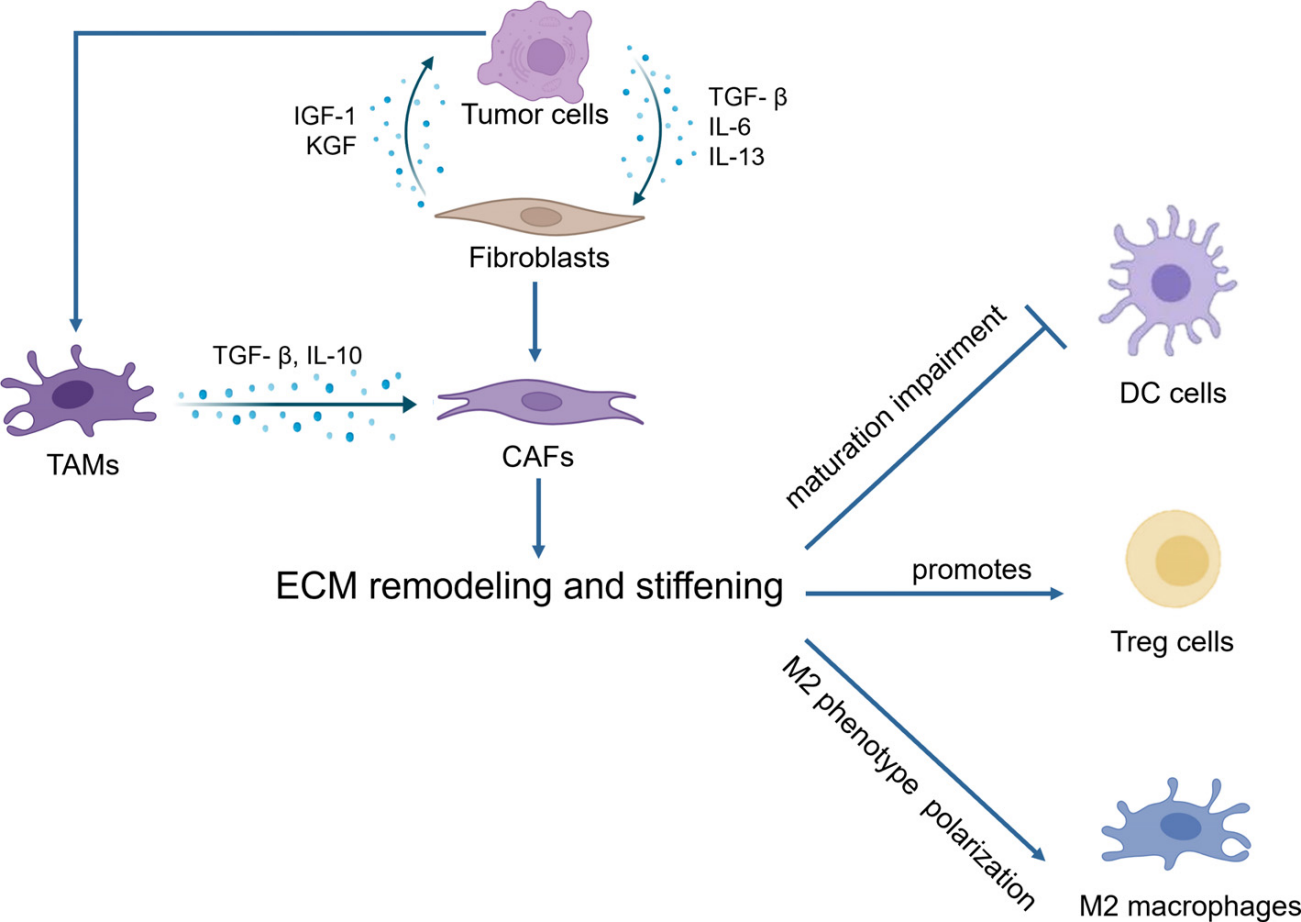
Immune cells utilize integrins to facilitate interactions with cell adhesion molecules.

Immune cells utilize integrins to facilitate interactions with cell adhesion molecules, which is essential for communication with other cells and the ECM (**Figure 2**). Notably, the integrin α4β1 acts as the primary receptor for VCAM-1 on leukocytes (171). Moreover, α4β1 and αxβ2 can collaborate to bind VCAM-1, significantly enhancing leukocyte adhesion (172). Additionally, macrophage integrins α4 and α9 are pivotal in promoting both macrophage migration and survival (173). On the other hand, the activation of integrin αVβ3 in macrophages can sustain chronic inflammatory responses in pathological conditions. Conversely, the loss of αVβ3 ligation allows macrophages to exit the inflammatory state, highlighting its role in inflammation modulation (174).

Integrins also play a vital role in T cell functionality. For instance, blocking αvβ6 can inhibit SOX4 expression and enhance T cell-mediated cytotoxicity in response to immune checkpoint inhibitors, particularly in triple-negative breast cancer mouse models (175). Furthermore, integrin αvβ8 is predominantly expressed in CD4^+^CD25^+^ T cells within tumors. The specific deletion of β8 from T cells can mitigate TGFβ-mediated inhibition of CD8^+^ T cells, thereby restoring their tumor-killing capacity and synergizing with immunotherapies (176).

Monoclonal antibodies, such as LM609, are among the first integrin antagonists to be developed and have been reported to have antiangiogenic effects in preclinical models (177). Its humanized version, etaracizumab (MEDI-522), is one of the first integrin antagonists to enter clinical trials because of its efficacy in preclinical studies and has completed a phase II clinical trial in malignant metastatic melanoma (178) (NCT00066196). The human Avintegrin-specific monoclonal antibody CNTO95 against the αVβ3 and αVβ5 integrins exhibited good safety in phase I and II clinical trials and demonstrated antitumor activity (179–181) (NCT00888043, NCT00246012). CNTO95 and etaracizumab are being evaluated in further clinical trials. Cilengitide, an inhibitor of the αVβ3 and αVβ5 integrins, has completed phase II trials in patients with recurrent prostate cancer (182) (NCT00103337, NCT00121238) and NSCLC (183) (NCT01118676, NCT00842712) and is currently undergoing phase II and III trials in glioblastoma (184, 185) (NCT00813943, NCT00093964, NCT00689221). In nonclinical studies, αVβ6 integrins have been shown to inhibit the activation of TGF-β in nonclinical studies (186). GSK3008348, a small molecule inhibitor of αVβ6 integrin and the first inhaled compound of this class of drugs, is safe and well tolerated by inhalation administration. A phase I clinical trial of GSK3008348 for the treatment of idiopathic pulmonary fibrosis has been completed (187) (NCT03069989, NCT02612051).

#### 5.2.2 FAK

**Figure 3:**
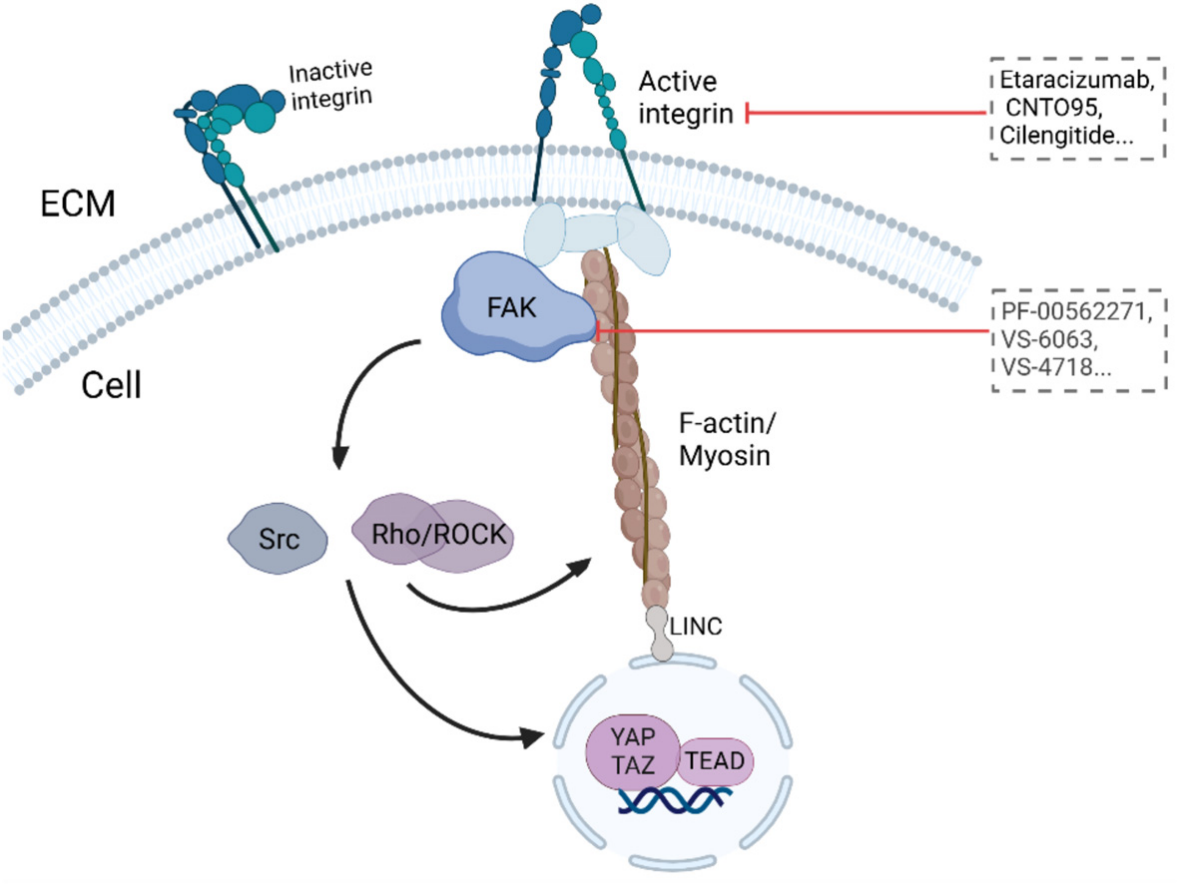
Targeting ECM stiffness for improved cancer therapy.

FAK, a cytosolic nonreceptor tyrosine kinase, is activated by integrin clustering and functions as a key regulator of focal adhesion dynamics (**Figure 3**, **Table 2**) (188). It plays a critical role in cellular responses to ECM stiffness, making it a promising target for inhibiting such mechanotransduction pathways (189). The phosphorylation of FAK is increased in response to increased matrix stiffness, with constitutive phosphorylation observed in myofibroblasts (188, 190). In addition, our previous data revealed that FAK inhibition alters the fibrotic and immunosuppressive TME in pancreatic cancer and renders tumors responsive to immunotherapy (191). As such, FAK is also a potential target to mediate matrix stiffness responses.

The first FAK inhibitor, PF-562271, was tested in a phase I clinical trial with tolerable results and controllable safety. A total of 99 patients with advanced malignant tumors were treated with PF-562271. After treatment, two-thirds of the patients were stable (approximately 6 weeks after the end of cycle 2). This first clinical trial revealed FAK as a promising therapeutic target (192) (NCT00666926). The FAK inhibitor VS6063, acquired by Verastem, has good pharmacodynamic characteristics (192) and has completed a phase I clinical trial in combination with paclitaxel in patients with advanced ovarian cancer (193) (NCT01778803). The inhibitors VS-4718 and VS-5095 also effectively target FAK kinase activity. Furthermore, the VS-4718 inhibitor is currently in clinical trials for patients with metastatic nonhematological malignancies (NCT01849744). The recently developed FAK inhibitor GSK2256098 has also been tested in clinical trials (194, 195) (NCT01138033, NCT02428270) and has completed phase II clinical trials in pancreatic cancer (194) (NCT02428270).

The FAK inhibitors examined in clinical trials to date have controlled toxicity and good safety and have shown extended progression-free survival as monotherapy inhibitors without clinical or radiological effects. Trials are underway to increase the efficacy of treatment by combining FAK inhibitors with cytotoxic chemotherapy, targeted therapy or immunotherapy.

## Conclusion

While the biological signals within the TME have been well studied, the specific physical cues and mechanisms of mechanical signals remain unclear. This article discusses the impact of mechanical factors, particularly the stiffness of the matrix, on the tumor immune microenvironment. Furthermore, we explored potential targets for modifying the stiff TME. By illuminating these concepts, our goal is to raise awareness about the crucial role of the physical environment in cancer and offer strategies to manipulate the TME to improve cancer therapy outcomes.
